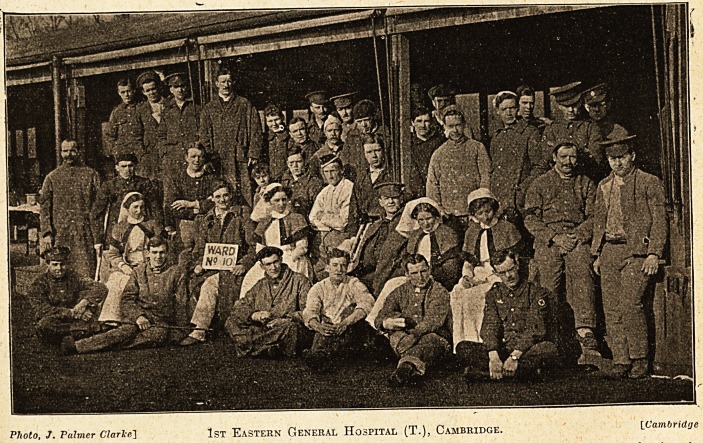# First Eastern General Hospital (T), Cambridge

**Published:** 1916-12-16

**Authors:** 


### FIRST EASTERN GENERAL HOSPITAL (T), CAMBRIDGE.

The illustration given below, which was taken twelve months ago, represents one of the open-air wards in the 1st Eastern General Hospital, about which so much has been written and so many diverse opinions have been expressed. The illustration indicates that the soldier patients were doing extremely well in the open-air wards, and it is fair to conclude that those in each ward and the sister and nurses formed a well-disciplined, friendly family. We have never regarded this as one of the most successful attempts in temporary hospital construction which the war has produced. There were palpable errors in the planning of the original pavilions, the most noticeable being the narrow passage-way which extended practically through the majority of the ward blocks, and was the cause of no little discomfort, and worse sometimes, we are by all the nursing staff alike. When the Officer Commanding eighteen months ago had an offer from a friend to erect a reading and resting room in the hospital grounds for the nurses, the latter unanimously decided against the proposal. When off duty the nurses have the best of accommodation, two large suites of rooms in the Fellows' building at King's College having been given up to their use. The nurses have been very happy at King's College, we learn, and have organised various debating and social clubs for their own recreation during the winter months. In summer-time tennis courts in the King's Fellows' garden on the hospital site have been placed at the nurses' disposal.

The nurses' commissariat is provided for, under the supervision of a catering committee of nurses, by the custodian of the pavilion on the hospital

*Photo, J. Palmer Clarke*] 1st Eastern General Hospital (T.), Cambridge. [*Cambridge*

afraid, when a certain type of patient had to be moved on a stretcher.

The war has made members of the nursing profession acquainted with very varying types of accommodation, from the excessively comfortable to some of the roughest and least inviting quarters to be found in temporary hospitals and old buildings suddenly opened for the reception of patients and the hospital staff. At the 1st Eastern General Hospital the nurses had no adequate provision or privacy when on duty. It is the view of the authorities that nurses who are constantly employed, as at this hospital, do not require rest whilst on duty, spare moments being occupied by preparation and the re-rolling of bandages. The sisters, too, had no spare room in connection with the wards, and the small hut or ward which was available was used ground and his brother. We have authority for saying that frequent inspection and observation justify the conclusion that the meals supplied to the nurses are excellent. No distinction is made in the food provided for the matron, sisters, nurses, Red Cross and V.A.D. staff attached to the hospital.

This hospital was mobilised on August 5, 1914, the first patient being received on the 15th of that month; 68 officers and 17,741 men were admitted during the first fifteen months. Of these, forty per cent. of the cases of cerebro-spinal meningitis admitted died. We have not received later figures, and the report of the Principal Medical Officer was satisfactory.

This is a hospital which everyone, who is interested in temporary hospital construction, should visit and carefully inspect.

## Figures and Tables

**Figure f1:**